# A procedure for in vitro evaluation of the immunosuppressive effect of mouse mesenchymal stem cells on activated T cell proliferation

**DOI:** 10.1186/s13287-021-02344-3

**Published:** 2021-06-05

**Authors:** Catalina-Iolanda Marinescu, Mihai Bogdan Preda, Alexandrina Burlacu

**Affiliations:** Laboratory of Stem Cell Biology, Institute of Cellular Biology and Pathology “Nicolae Simionescu”, 8 B.P. Hasdeu Street, 050568 Bucharest, Romania

**Keywords:** Mesenchymal stem cells, T cells, Immunosuppression, CFSE, Proliferation index, Nylon wool, Splenocytes, Cell culture, Flow cytometry

## Abstract

**Background:**

Mesenchymal stem/stromal cells (MSC) represent adult cells with multipotent capacity. Besides their capacity to differentiate into multiple lineages in vitro and in vivo, increasing evidence points towards the immunomodulatory capacity of these cells, as an important feature for their therapeutic power. Although not included in the minimal criteria established by the International Society for Cellular Therapy as a defining MSC attribute, demonstration of the immunomodulatory capacity of MSC can be useful for the characterization of these cells before being considered MSC.

**Methods:**

Here we present a simple and reliable protocol by which the immunosuppressive effect of mouse bone marrow-derived MSC can be evaluated in vitro. It is based on the measuring of the proliferation of activated T cells cultured in direct contact with irradiated MSC.

**Results:**

Our results showed that mouse MSC have a dose-dependent inhibitory effect on activated T cell proliferation, which can be quantified as a percentage of maximum proliferation. Our data shows that batch-to-batch variability can be determined within one or multiple experiments, by extracting the area under curve of T cell proliferation plotted against the absolute number of MSC in co-culture.

**Conclusions:**

The validation of the immunosupressive capacity of MSC could be added to the characterization of the cells before being used in various MSC-based approaches to treat immunological diseases. Our results showed that mouse MSC have a dose-dependent inhibitory effect on activated T cell proliferation. The immunosuppressive properties of MSC vary between batches, but not between different passages of the same batch.

## Background

Mesenchymal stem/stromal cells (MSC) represent adult cells with multipotent capacity. They were historically isolated from bone marrow; however, their abundance in the adipose tissue had been largely documented thereafter. Besides the two major sources of MSC, a lot of other organs have been acknowledged to host such cells, which make their identity sometimes disputable [1–3].

According to International Society for Cellular Therapy (ISCT), MSC are defined as spindle-shaped plastic-adherent cells, which are negative for hematopoietic markers (CD45, CD14, CD11), positive for several stromal markers (CD105, Sca-1, CD29, CD44), and have the capacity to differentiate into osteoblasts, chondroblasts, and adipocytes, when cultured under appropriate conditions [4]. These cells can generally orchestrate tissue repair by (1) cellular differentiation, (2) direct immune-modulation, (3) production of growth factors that drive neovascularization and cytoprotection, and (4) communicating with resident cells to stimulate tissue repair [5].

Increasing evidence points towards a broad immunomodulatory capacity of these cells, as an important feature that can explain the therapeutic power of these cells [6–8]. Based on this quality, MSC are increasingly used in clinic in bone marrow transplants to cure important diseases with immune component, such as autoimmune diseases, graft-versus-host disease and allograft rejection [9, 10]. Their pleiotropic immunomodulatory properties also make them appropriate candidates for other diseases, such as cardiovascular diseases and wound healing, in which the secreted molecules positively impact the inflammatory phase of the healing process, favoring reduced inflammation and increased regeneration and healing [11].

A particular feature of MSC is that their immunoregulatory fate can be converted based on the dynamics of the inflammatory process, the strength of immune system activation, and the local cytokines. This particularity makes MSC good candidates for ameliorating tissue damage in the pathology of nearly all organs in the body [12]. Thus, MSC are capable of influencing both adaptive and innate immune responses and have a dual immunomodulatory capacity: they have the ability to inhibit immune cell activation and proliferation and also to enhance immune responses via the secretion of pro-inflammatory factors and chemokines [13–17]. The nature of the immunomodulatory effect of MSC in living organisms is complex and depends on a multitude of local factors and conditions, as well as on the context of the disease microenvironment [12].

The ability of MSC to adopt a different phenotype in response to sensing the inflammatory environment is important for their therapeutic potential, yet it is not captured in assays that are commonly used to characterize these cells [5, 13]. Particularly, although not included in the minimal criteria established by ISCT as a defining MSC characteristic, the estimation of the immunosuppressive potential of the cells can help characterize these cells before being considered MSC. This immunosuppressive property of MSC should be quantified by standardized immune assays with purified cells as responders, as already proposed several years ago [18].

Here we present a standardized and reliable protocol by which the immunosuppressive potential of mouse bone marrow-derived MSC can be evaluated in vitro. This protocol is based on the assessment of the proliferation index of activated T cells in direct interaction with irradiated MSC in culture and can be useful for determining the variability among different MSC batches and for predicting their in vivo efficacy.

## Methods

### Animals

C57Bl6/J mice were purchased from the Jackson Laboratory and bred in the local animal facility. Mice were maintained under specific pathogen-free conditions in a controlled environment with a 12/12-h light/dark cycle, 21°C, and 55–60% humidity and had access to chow and water ad libitum. Mice were euthanized by cervical dislocation before spleen and bone collections. All experiments were conducted in accordance with the European Guidelines for Animal Welfare (Directive 2010/63/EU) and approved by the National Sanitary Veterinary and Food Safety Authority (nr 296/23.08.2016).

### Materials

CFSE (C1157) and CD3/CD28 beads (11452D) were purchased from ThermoFisher Scientific. The following antibodies were purchased from Biolegend: anti-CD3-PE (100206), anti-CD4-PB450 (100438), anti-CD8-APC/Cy7 (100765), anti-B220-FITC (103206), anti-CD45-PE (103106), anti-Sca-1-PE (108108), anti-CD73-PE/Cy7 (127224), and anti-CD105-PB450 (120412). The following antibodies were purchased from R&D Systems: anti-CD29-PE (FAB2405P), anti-CD90-PE (FAB7335P), and anti-CD44-PE (FAB6127P).

### MSC isolation and characterization

**Fig. 1:**
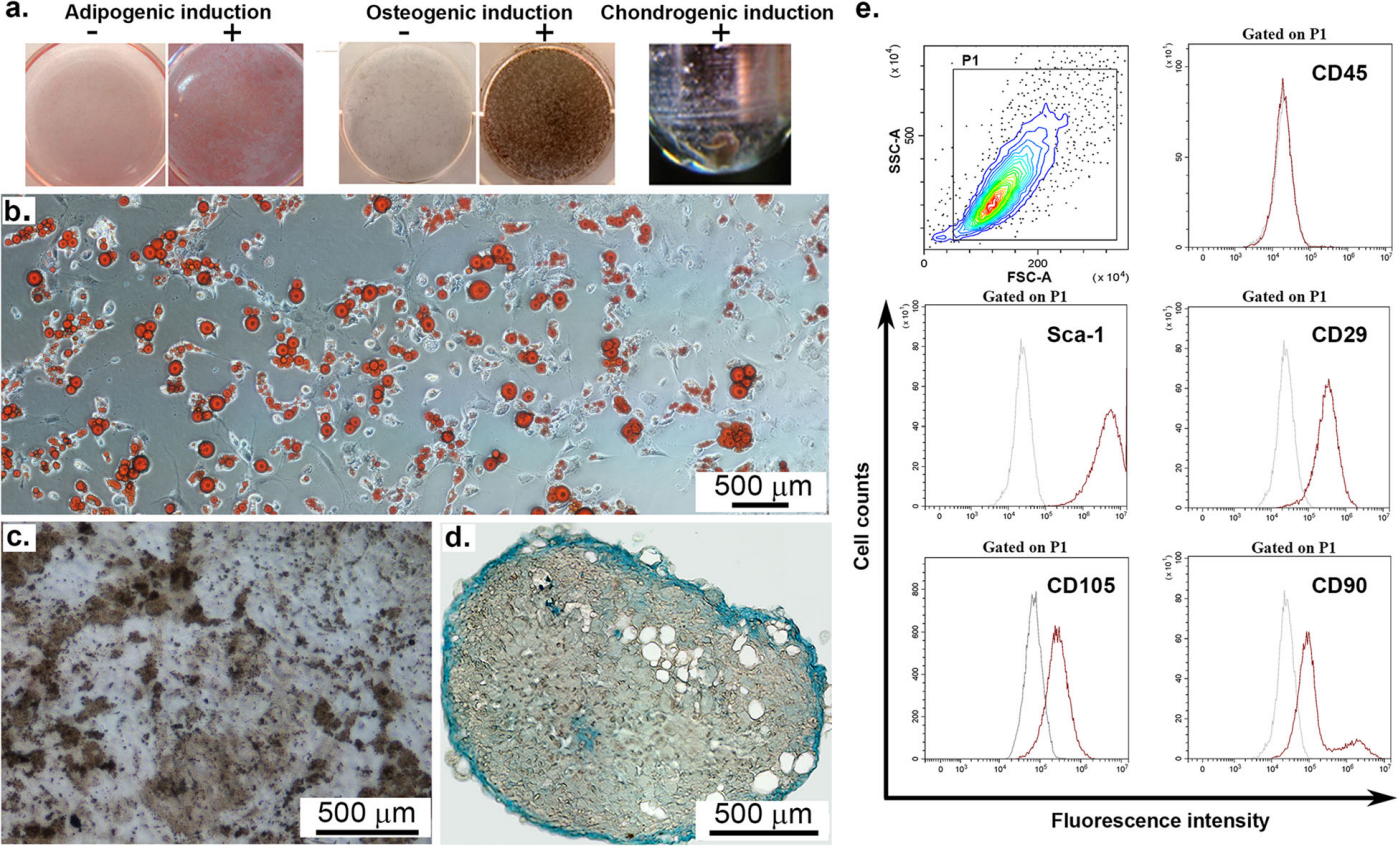
MSC characterization in accordance to International Society for Cellular Therapy. **a** Adipogenic, osteogenic, and chondrogenic differentiation of MSC, as comparisons between cells cultured in the presence of appropriate differentiation factors and normal culture medium (non-differentiating factors). Exception is chondrogenic differentiation, for which no pellet was formed in the absence of chondrocyte-specific differentiation medium. **b**–**d** Representative fields of differentiated MSC by Oil red (**b**), von Kossa (**c**), and Alcian Blue (**d**) staining, demonstrating the capacity of MSC to generate adipocytes (**b**), osteoblasts (**c**), and chondroblasts (**d**), respectively, when cultured in appropriate differentiation media for three weeks. **e** Phenotypic characterization of MSC by flow cytometry, illustrating the lack of CD45 expression and the expression of Sca-1, CD29, CD105, and CD90

Four MSC batches were obtained from bone marrow aspirate of male 6-week-old C57Bl/6 mice as previously described [19]. Briefly, the bone marrow aspirate, obtained by flushing the medullar channels of tibiae and femurs from one mouse with 5 ml culture medium (DMEM containing 10% MSC-qualified FBS), was gently passed through needles with increasing gauges (from 21 to 25) in order to generate a single cell suspension. The volume of cell suspension was adjusted to 10 ml and the cells were directly seeded on two 60-mm diameter tissue culture-treated Petri dishes and incubated at 37°C under 5% CO2 atmosphere in culture medium. The first two passages were performed at around 7-day intervals, by using 0.25% trypsin and gentle scraping with the rubber policeman. Starting from the third passage, the cells were detached at 80% confluence by 2-min trypsinization and replated on 0.1% gelatin-coated plates at 5000 cells/cm^2^. Cells were used between 6th and 12th passages. MSC were routinely characterized for the presence of specific surface markers (by flow cytometry) and for the potential to generate adipocytes, osteoblasts, and chondroblasts, when cultured under appropriate conditions (Fig. 1). Flow cytometry analysis demonstrated the absence of the hematopoietic cell marker CD45 and the presence of surface antigens usually associated to MSC (Sca-1, CD105, CD29, and CD90). Adipocytes, osteoblasts, and chondroblasts were evidenced at 3 weeks after differentiation induction by Oil Red O staining of lipid droplets (Fig. 1a), von Kossa staining of calcium deposits (Fig. 1b), and Alcian Blue staining of acid mucopolysaccharides (Fig. 1c), respectively.

### Assessment of the immunosuppressive potential of MSC

The procedure has three major steps, which are to be performed in different days:

- Step 1 (Day 0; ~2 h): Mitotic inactivation of MSC followed by seeding on tissue-culture wells. Around 1.5 × 10^5^ cells are enough for the procedure. This step is performed 1 day before the interaction of MSC with T cells.
- Step 2 (Day 1; ~ 6 h): Isolation of splenic T cells and fluorescent labeling with Carboxy-fluorescein succinimidyl ester (CFSE) before the addition onto MSC culture in the presence of CD3/CD28 activation beads.
- Step 3 (Day 4, ~ 4 h, including flow cytometry analysis): Assessment of the proliferation index of T cells after 3 days in culture, based on CFSE dye dilution assay.

### MSC irradiation

MSC were detached with 0.25% trypsin and re-suspended at a density of 10^5^ cells/ml in MSC complete medium (DMEM low glucose, supplemented with 10% MSC qualified FBS, in the presence of 1% antibiotic-antimycotic solution). Cell irradiation was performed with a RS-2000 X-ray irradiator at 30 Gy dose, in 1.5-ml microcentrifuge tube. We routinely irradiated the tubes in the middle of the third shelf (for an evenly radiation distribution) for 16 min and 36 s. After irradiation, cells were seeded in 96-well plate wells, using serial dilutions ranging from 10,000 to 625 cells/well. Technological triplicates were used for each MSC dilution. The irradiated cells were placed in the incubator under 37°C and 5% CO_2_ atmosphere and left overnight before interaction with T cells.

### Isolation of mouse splenocytes

Mice were **s**acrificed by cervical dislocation and prepared for dissection by disinfecting the fur and skin with 70% alcohol. The peritoneum was exposed through a midline incision of the skin. The peritoneal wall was cut along the linea alba and then laterally under the ribcage, towards the left side of the mouse. The bowels were gently grabbed and pulled aside to expose the spleen, which was detached (by cutting the connections with other tissues) and introduced into a 15-ml tube containing 2 ml ice-cold PBS/FBS (PBS supplemented with 2% FBS). Under the laminar flow hood, the tube content (spleen and PBS/FBS) was transferred into a 70-μm cell strainer adapted on the top of a 50-ml tube. The spleen was gently triturated (without cutting in pieces), using the thumb head of a 5-ml syringe plunger, while adding 10 ml of PBS/FBS, and cell pellet was resuspended in 5 ml ice-cold red blood cell lysis buffer and incubated at 24°C for 4 min. The lysis was stopped by adding 10 ml ice-cold PBS/FBS and centrifugation at 400×*g*, 4 °C, 10 min. Cells were resuspended in 2 ml complete RPMI medium (RPMI ATCC-formula, supplemented with 10% FBS and 50 μM β-Mercapto-ethanol), counted and used in T cell enrichment step. An aliquot of around 20 μl (~10^6^ cells) was saved for flow cytometry analysis of splenocytes to detect the percentage of B and T cells, as well as the percentages of CD4^+^ and CD8^+^ cells within T cell population. Briefly, 100 μl cell suspension containing 10^5^ cells was incubated with fluorescent-labeled antibodies specific for B220, CD3, CD4, and CD8. After 30 min of incubation at 4°C, the cells were washed by centrifugation and resuspended in FACS buffer (PBS containing 2% FBS) for flow cytometry analysis. Propidium iodide (2 ug/ml final concentration) was added before analysis to identify the live cells and at least 30,000 events were considered for each sample. Acquired data was analyzed using CytExpert software (Beckman Coulter). The compensation matrix was obtained using compensation beads (Thermo Fisher Scientific) combined with fluorescent antibodies for every single-color sample.

### T cell enrichment from splenocytes using the nylon wool column

The protocol described by Hathcock, with a few minor changes, was used with good results [20]. The nylon wool column was prepared in advance by inserting 1g of nylon wool (Kisker Biotech - MKN-100) into a 10-ml sterile syringe and autoclaving at 110 °C for 15 min. Briefly, T cell enrichment followed these steps: (1) the column was placed on the stand and a 3-way stopcock with a 19-G needle was attached to the column; (2) with the stopcock in open position, the column was washed 3 times with 8 ml warm RPMI complete medium. During the first wash, the wool was firmly pressed with a 10-ml sterile serological pipette to remove the air bubbles; (3) the stopcock was closed, then 2 ml warm complete RPMI medium was added to the column and the column was allowed to equilibrate in the incubator for at least 45 min; (4) the column was moved back in the cell culture hood, the stopcock was opened, and the column was let to drain completely; (5) the 2-ml splenocyte suspension was carefully layered on the column surface (not on the wall of the syringe) and the suspension was allowed to drain completely; (6) the column was washed with 0.5 ml warm complete RPMI medium to ensure that all cells had penetrated the wool; (7) the stopcock was closed, and 2 ml warm complete RPMI medium was added onto the column before being placed in the incubator for another 45 min; (8) the column was removed from the incubator, the 19G needle was carefully replaced with a 23G needle, the stopcock was opened, and T cells were eluted by washing the column four times with 10 ml warm complete RPMI medium; (9) the eluate (~40 ml) was centrifuged at 400×*g*, 24 °C, 10 min, and then the cells were resuspended in 1 ml warm medium. An aliquot of 50 μl was saved for counting (with an intermediate dilution of 1/50) and flow cytometry analysis. The remaining cells were washed with 10 ml PBS/FBS and centrifuged at 400×*g*, 24 °C for 10 min. At the end, the cells were resuspended in 950 μl PBS +1% FBS and transferred in a 15-ml conical tube.

### T cell labeling with CFSE

Uniform labeling of T cells is important for accurate determination of cell proliferation. Our protocol followed the next steps: (1) First, a vortex was brought in the hood and 50 μl CFSE 100 μM was freshly prepared in PBS with 1% FBS (from a concentrated stock solution); (2) while keeping the tube with 950 μl cell suspension in a 45° position, the 50-μl CFSE solution was carefully placed as a stable drop on the upper part of the wall inside the 15-ml tube, making sure that the cell suspension and CFSE solution did not make contact; (3) the vortex was turned on, then the lid of the tube was closed, and the tube was immediately turned upside down and vortexed for 1–2 s to ensure the rapid and homogenous dispersal of CFSE solution (the final concentration of CFSE was 5 μM); (4) the tube was then incubated at 24°C in the dark for 10 min; (5) the 15-ml tube was filled up with complete RPMI medium and centrifuged at 400×*g*, 4°C, 10 min; (6) the supernatant was decanted and the wash procedure was repeated for another 2 times with 10 ml complete RPMI medium; and (7) the cell pellet was resuspended in complete RPMI medium at a cell density of 10^6^ cells/ml for plating. An aliquot of 200 μl (around 2 × 10^5^ cells) was saved for flow cytometry validation of the homogenous staining.

### Co-culture of T cells with the irradiated MSC

The immunosuppressive effect of MSC was evaluated by culturing T cells in the direct contact with irradiated MSC, in activating conditions. Different MSC to T cell ratios, within the range 1:10 to 1:160, were obtained by serial dilutions of MSC suspension, while keeping the number of T cells constant. The greatest number of MSC per well (10^4^ cells/well) was calculated so that to obtain a confluent layer of inactivated cells. Activation of T cells was done by mixing T cells with anti-CD3/CD28 microbeads in a 1:1 ratio (bead to T cell) in RPMI complete medium. Control wells important in this step: irradiated MSC only (to ensure that after 3 days in culture, irradiated MSC are healthy), activated T cells cultured without MSC (to determine the maximum level of T cell proliferation in culture), and T cells cultured in the absence of anti-CD3/CD28 beads (to establish the fluorescence median value of CFSE after 3 days in culture for accurate assessment of the proliferative index).

### Flow cytometry analysis

After 3 days of co-culture of T cells with MSC in cell-to-cell contact, all cells and beads in the wells were collected for flow cytometry analysis. To achieve this, the following steps were performed: (1) the supernatant of each well was collected in separate 1.5-ml microcentrifuge tubes; (2) the wells were washed with 100 μl PBS/well with recovering of the washed volume in the corresponding tube; (3) the cells were trypsinized by incubating each well with 30 μl Trypsin-EDTA solution (0.25% Trypsin mixed with 0.2 g/l EDTA-4Na in Hank’s Balanced Salt Solution) for 2 min, followed by trypsin inactivation with 100 μl complete RPMI medium and collection of whole volume in the corresponding tube; (4) each well was washed with an additional 100 μl PBS, and the washed volume was saved in the same corresponding tubes (the final volume in each tube was around 450 μl); (5) the tubes containing beads were placed in the magnet for 2 min, and the cell-containing medium was recovered in fresh tubes; (6) the beads were washed with 500 μl PBS/FBS by pipetting and re-placing the tubes into the magnet for another 2 min; the supernatant was recovered in the corresponding fresh tube (the total volume of cells was around 1 ml/tube); (7) the cells were centrifuged at 300×*g*, 4°C, 5 min and resuspended in 250 μl PBS/FBS; and (8) 2 μg/ml propidium iodide was added to each sample and cells were analyzed using a Beckman Coulter CytoFlex flow cytometer.

### ELISA

The supernatant media derived from the resting T cells, activated T cells, and MSC-T cell co-cultures were collected and the interferon (IFN)-γ was measured using a mouse IFNγ ELISA kit (R&D Systems) according to the manufacturer’s instructions.

## Results

### Characterization of T cells

**Fig. 2:**
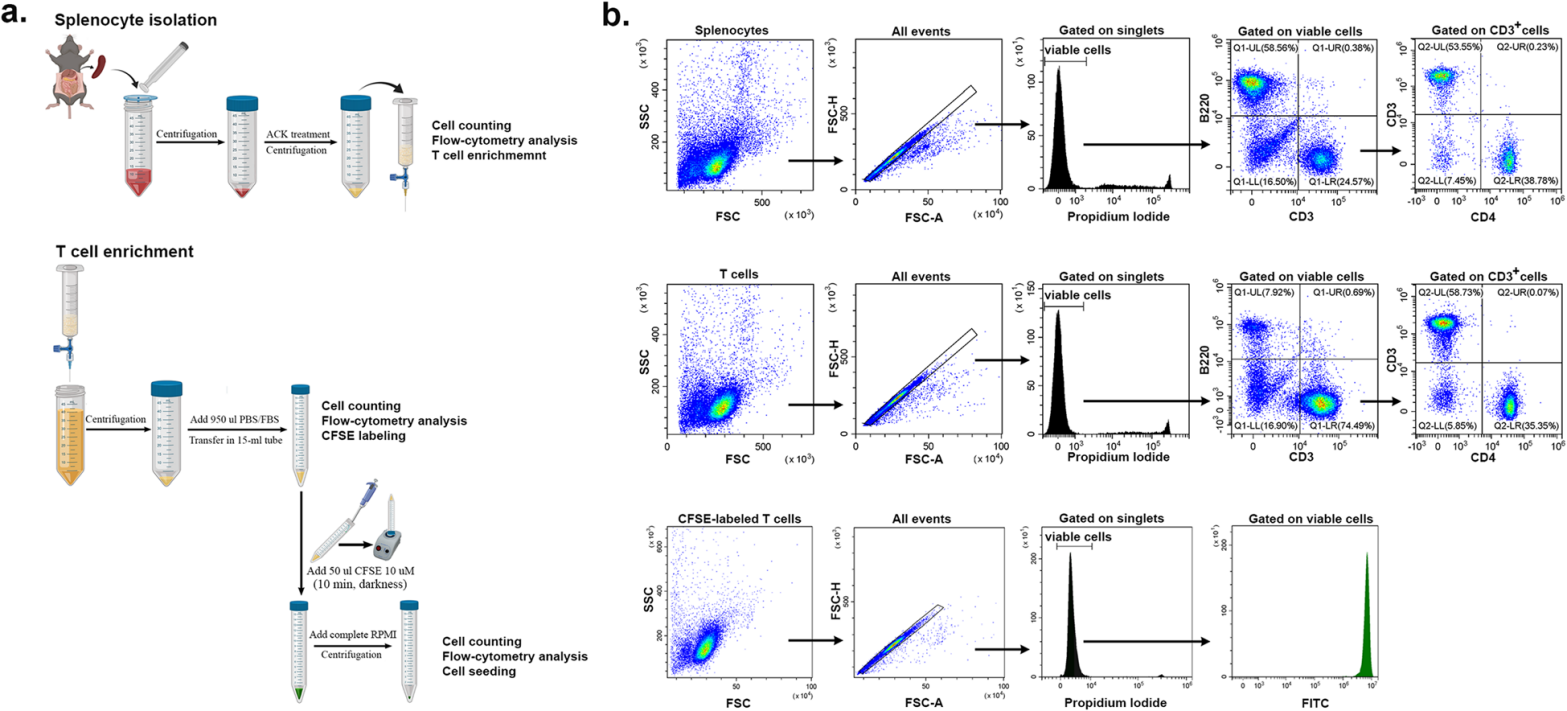
Schematic design of the main steps for T cell isolation and CFSE labeling. **a** Isolation of mouse splenocytes and T cell enrichment on nylon wool column. The critical step of CFSE staining of T cells is also shown. **b** Flow cytometry characterization of splenocytes (upper line), T cells (middle line) and CFSE-labeled T cells (lower line). Note the depletion in B220^pos^ cells in T cell enriched population, as compared to whole splenocyte population (~ 8% B cells after enrichment, as compared to ~ 60% in the initial population) and the relatively unchanged CD8:CD4 ratio after enrichment. Also note the sharp fluorescent peak of T cell population obtained after CFSE staining

Schematic illustration of the procedures used for splenocyte isolation and T cell enrichment on nylon wool is given in Fig. 2a, together with flow cytometry characterization of cells obtained after each step (Fig. 2b). Around 10^8^ splenocytes are obtained from one spleen of adult C57Bl/6J mouse, with a B cell to T cell ratio of more than 2:1 and a ratio of 1.5 CD8 cells to 1 CD4 cell within T cell sub-population. Our results showed that the percentage of T cells in T cell-enriched population was more than 3-fold increased as compared to that in the splenocyte population. The nylon wool treatment did not importantly alter the ratio between CD4^+^ and CD8^+^ cells in T cell population, as compared to splenocyte population. CFSE staining produced a uniformly stained population, which was demonstrated by the sharp peak on FITC fluorescence channel. Importantly, CFSE-labeled T cells at time *t*=0 is not to be used as the negative control for T cell proliferation, because part of the incorporated CFSE is lost during the 3-day culture, and consequently, the fluorescence of CFSE-labeled resting T cells after 3 days in culture is one order of magnitude lower than the fluorescence at time *t*=0.

### Co-culture of CFSE-labeled T cells with the irradiated MSC

**Fig. 3:**
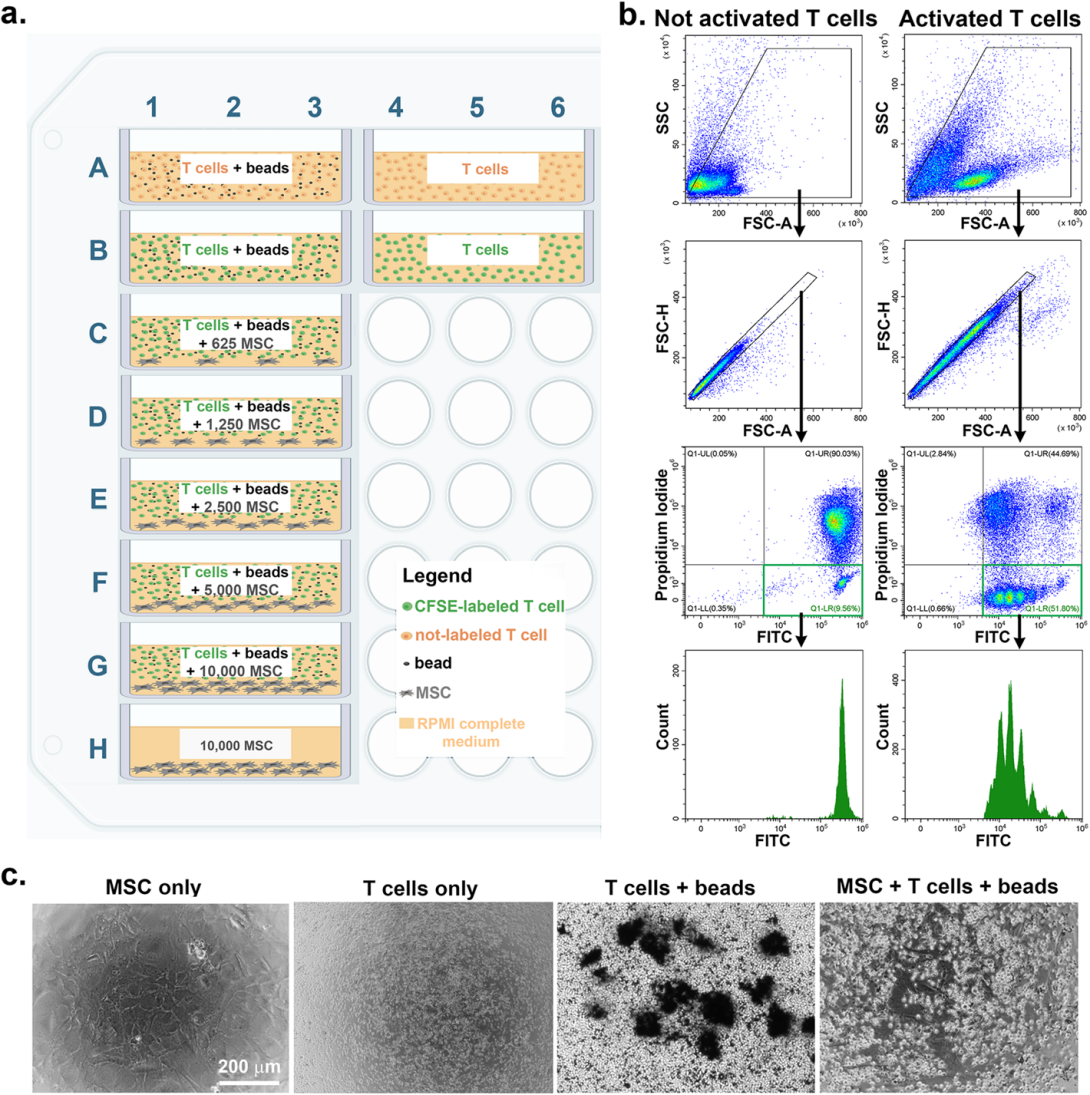
**a** The 96-well plate template for experimental design. Thirty wells are required for testing one MSC batch, of which 12 wells serve as controls (the first two lines of the plate). Each additional MSC batch will require another 18 wells, which can be aligned as triplicate columns after the first batch. A total of four different batches can be analyzed in one 96-well plate. **b** Flow cytometry analysis of the T cell population in the negative and positive controls, at the end of 3-day co-culture experiment. The gating strategy consists of removal of debris, doublets, and dead cells and analysis of the viable cells on the CFSE histogram. Multiple fluorescent peaks are distinguished in the positive control (activated T cells), as compared to the single peak in the negative control (not activated T cells). **c** Phase-contrast microscopy images of irradiated MSC, resting T cells and activated T cells in culture (in the presence or absence of MSC). Note the integrity of the confluent layer of irradiated MSC after 4 days in culture (MSC only) and the lower number of T cells in the presence of irradiated MSC (MSC +T cells + beads vs. T cells + beads)

The experimental set-up and the design of the 96-well plate wells needed for evaluation of one MSC batch is illustrated in the Fig. 3a. The wells include technological triplicates of five MSC dilutions (625, 1250, 2500, 5000, and 10,000 cells per well) and five controls (MSC only, not-labeled and CFSE-labeled T cells in resting conditions, and not labeled and CFSE-labeled T cells in activating conditions). T cell proliferation is evaluated by CFSE dilution after 3 days of culture using flow cytometry analysis, and the results are interpreted in relation to the negative (resting labeled T cells) and positive (labeled T cells cultured in activating conditions) controls. Gating strategy is illustrated in Fig. 3b, for the negative and positive controls. The population to be analyzed is selected by eliminating the debris (based on FSC and SSC scatter plot) and doublets (based on FSC-A vs. FSC-H), as well as dead cells (Propidium iodide-positive cells). The CFSE dilution is evaluated in the population of FITC^pos^/Propidium iodide^neg^ cells on the FITC histogram, where multiple fluorescence peaks are visible in the positive control, as compared to a single fluorescence peak in the negative control. One can note that the peak of not-activated T cells (the negative control) is one order of magnitude lower than the peak of labeled T cells at time *t*=0, with a median fluorescence intensity of 7×10^5^ after culture *vs* 7×10^6^ before culture (Figs. 2b and 3b). The phase contrast microscopy of cells in co-culture is illustrated in Fig. 3c. The data show that irradiated MSC maintained the viability and appeared as a confluent monolayer of cells after 4 days in culture. Furthermore, the proliferation of T cells is clearly visible in activating conditions (T cells+ beads), in comparison to not activated T cells (T cells only) and activated T cells in the presence of MSC (MSC+ T cells+ beads).

### A dose-dependent inhibitory effect of MSC on T cell proliferation

**Fig. 4:**
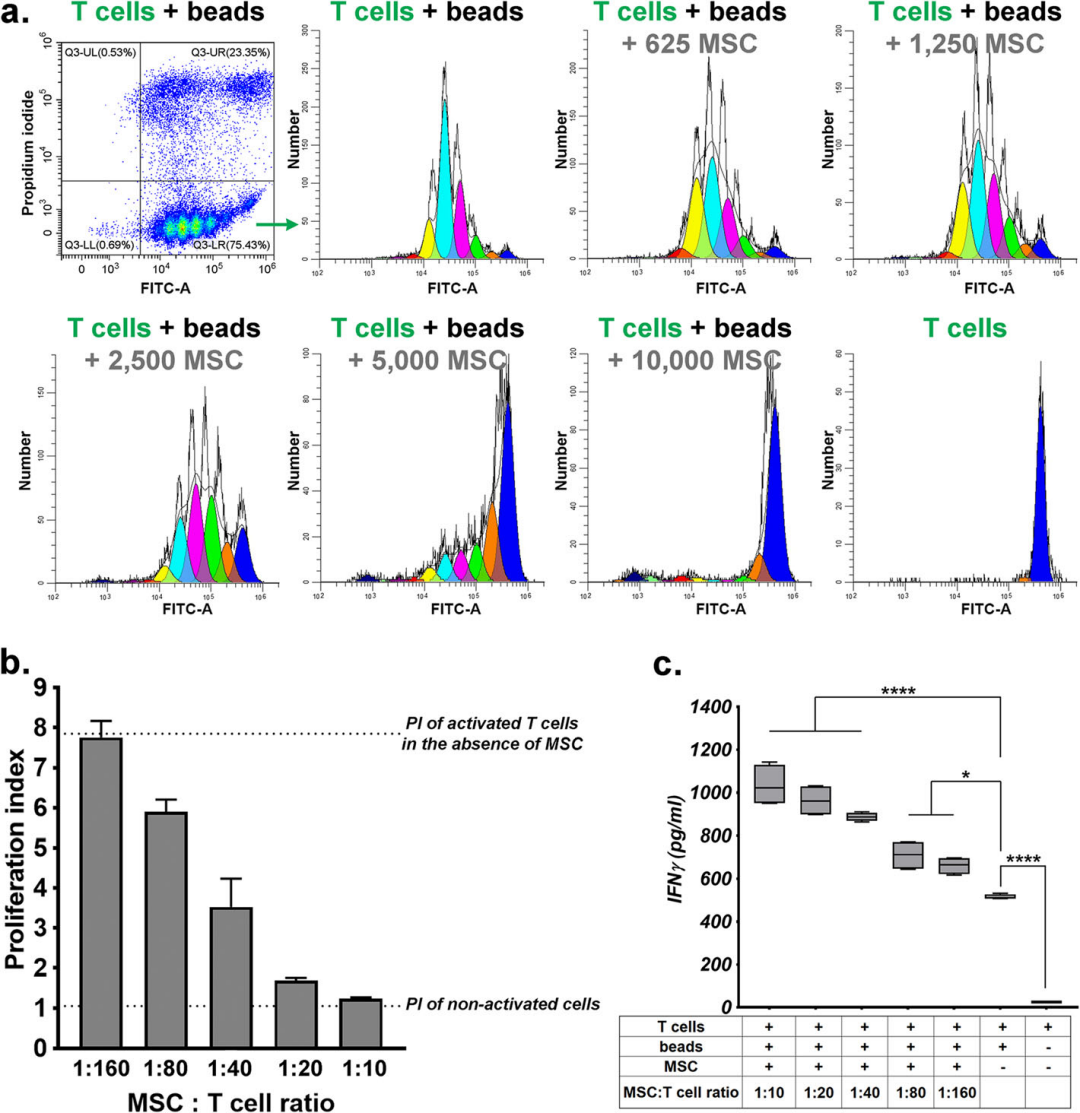
**a**, **b** T cell proliferation in the presence of MSC. **a** Histograms illustrate the CFSE fluorescent peaks of T cell samples activated in the presence of increasing amount of MSC. Note the single fluorescent peak in resting T cells, representing the parent population. Also note the diminishing of the parent population peak with the increasing number of MSC in the culture, concomitant with the increasing in the magnitude of the low-fluorescent peaks, as a result of dye dilution with each cell proliferation. **b** The diagram shows the decrease of the proliferation index (PI) of activated T cells in the presence of MSC in a dose-dependent manner. The data illustrate mean ± SD of one representative experiment performed in triplicate. **c** ELISA quantification of IFNγ in the secretome of co-culture. The data represent the mean ± SD of one experiment performed in quadruplicates with two MSC batches

The proliferation is being reported as proliferation index, which can be calculated using ModFit LT 5.0 software (Fig. 4a, b). Usually, lymphocyte proliferation can be monitored by flow cytometry for up to eight divisions before CFSE fluorescence is decreased to the background fluorescence of unlabeled cells [21]. The parent population is set based on the peak of the negative control (“T cells” sample in Fig. 4). This peak gradually diminishes as the cells proliferate in culture; the lower the parent population peak, the higher the proliferation rate. The results show a dose-dependent inhibition of T cell proliferation induced by the presence of MSC. By using increasing MSC to T cells ratios, a large range of inhibition can be achieved, which covers the whole interval between the positive and negative controls. Thus, total inhibition of T cell proliferation was obtained when MSC were present in a ratio of around 1:10 (MSC to T cells), while minimal inhibition was achieved with a 1: 160 (MSC to T cell) ratio. This data demonstrates strong immunosuppressive effects of MSC on activated T cells in culture.

Previous studies showed that activated T cells produced cytokines within 4–6 h and cytokine production was then maintained as long as the cells remained in direct contact with the antigen [22]. To evaluate T cell functionality in the presence of MSC, IFNγ was determined in the supernatant of the 3-day co-culture by ELISA. The results showed no IFNγ in resting T cell-derived medium. However, an increased accumulation of IFNγ levels was noted in the secretome of activated T cells, both in the absence and presence of MSC (Fig. 4c). Of note, higher levels of IFNγ were produced by activated T cells in co-culture with MSC than by T cells alone, which were directly correlated to the number of MSC used in co-culture. This result suggested a direct effect of MSC on stimulating IFNγ production at early stages of the co-culture before suppressing activated T cell proliferation and also highlighted the importance of the crosstalk between MSC and immune cells in mediating the immunosuppressive effects of MSC.

### MSC irradiated versus non-irradiated

**Fig. 5:**
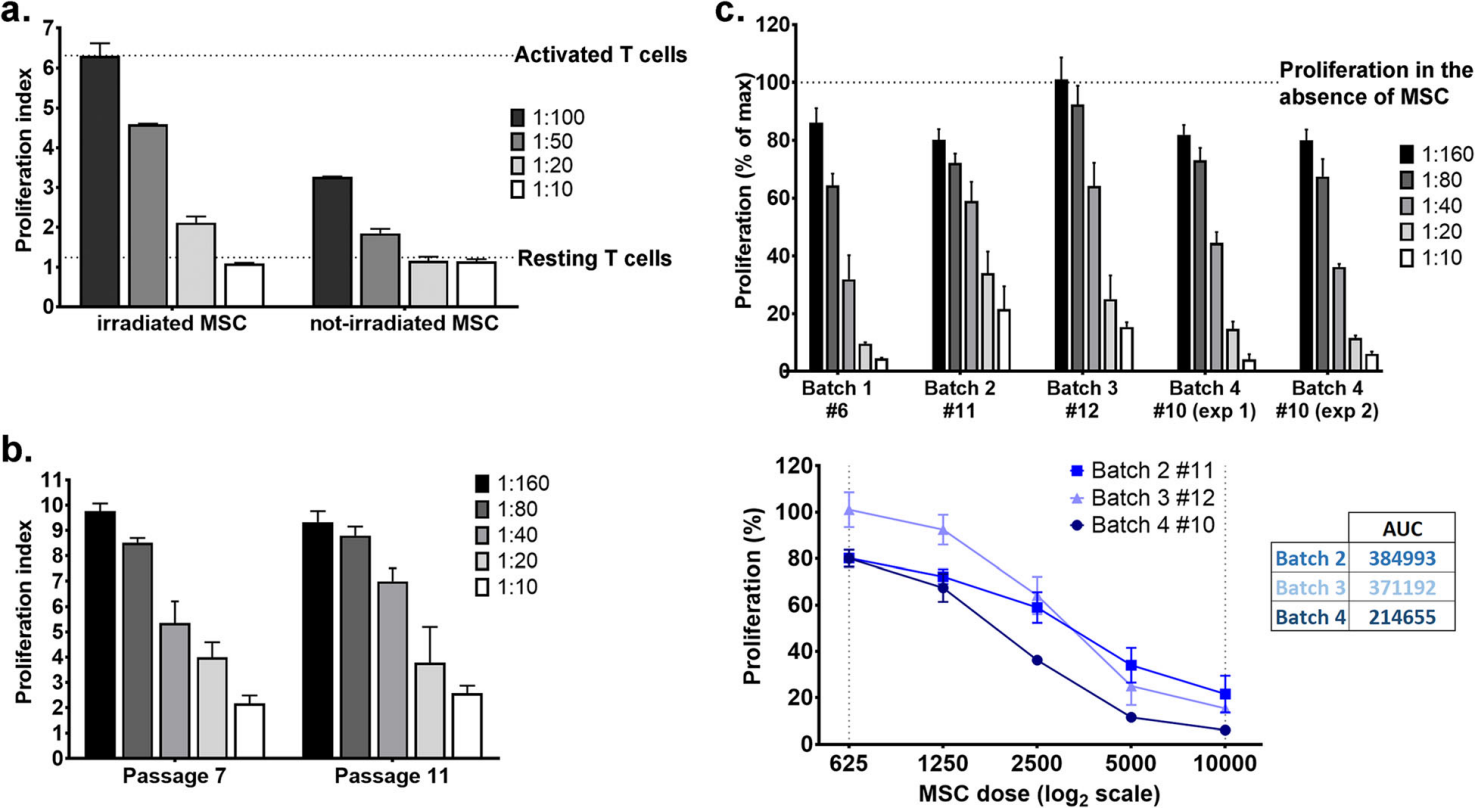
Dose-dependent inhibition of T cell proliferation by MSC. **a** The comparative illustration of the effect of actively proliferating and irradiated MSC on T cell proliferation. Note the loss of the dose-dependent effect of proliferative MSC on the inhibition of T cell proliferation. **b** Comparative analysis of the immunosuppressive effect of a batch of MSC at a low and a higher passage. Note that these MSC properties are not modified with increasing the passage number. The experiment represents one representative experiment from at least 6 experiments performed with different MSC batches. **c** Comparative analysis of the immunosuppressive properties of 4 batches of MSC measured in separate experiments. The comparison was done by reporting the proliferation as percentage (± SD) of maximum proliferation obtained in individual experiments. By plotting the proliferation against the dose of MSC, the area under curve can be calculated, which makes possible the comparison between the immunosuppressive properties of different batches. The lower the AUC, the higher the immunosuppressive effects

The mitotic inactivation of MSC is crucial in order to obtain a reliable dose-dependent effect on T cell proliferation. Figure 5a illustrates the loss of the dose dependence of the MSC inhibition when proliferating MSC are used in co-culture. Expectedly, higher inhibition was achieved with actively proliferating MSC, as compared to mitotically inactive MSC. The difference was more pronounced for lower doses of MSC as compared to higher doses, an effect that can be explained by the alteration of T cell to MSC ratio during co-culture, as a direct consequence of increasing the MSC number. In our hands, the mouse bone marrow-derived MSC have a population doubling time of around 50 h, between 6th and 14th passages, which would allow for almost 2 population doublings during a 4-day culture period.

### Applications of the methods

As MSC have strong and dose-dependent immunosuppressive effects of T cells in vitro, differences between MSC batches, passages, or conditions can be evaluated within the same experiment, by direct comparisons. Our data showed that no differences in the immunosuppressive properties existed between MSC at passages 7 and 11 of the same batch (Fig. 5b). Comparisons between different experiments can also be obtained by reporting the results as percentage of maximum proliferation. The normalization is required when data from different experiments are to be compared, due to the variation of PI value of activated T cells between different experiments, which also affect the percentage value of resting T cell proliferation in each individual experiment. For such comparisons, the maximum proliferation in each individual experiment was obtained by subtracting the PI value of resting T cells from the PI value of activated T cells. Figure 5c illustrates the comparative analysis of the immunosuppressive properties of four MSC batches obtained in separate experiments. All batches have inhibitory effects of T cell proliferation in culture. One batch (batch 4 at passage 10) was analyzed two times in independent experiments (performed in different days) and the results confirmed the reliability of the method (Fig. 5c). Comparisons between cells evaluated in different experiments can be done by plotting the T cell proliferation against the absolute number of MSC in co-culture (represented as log_2_scale) and calculating the area under the curve (AUC). Thus, our results revealed differences in the immunosuppressive capacity of various MSC batches, with batch 4 showing stronger inhibitory effects on T cells than the other batches.

## Discussion

### Starting material

Mouse MSC are most frequently isolated from bone marrow or adipose tissue. They are selected based on the adherence to the culture substrate and, usually, require several passages for depletion of contaminating hematopoietic cells, if no other methods of selection are chosen. Over the years, several protocols for mouse bone marrow MSC isolation were evaluated in our laboratory [19, 23, 24]. Particularly, in the case of C57Bl/6 mouse strain, which is reportedly the most popular mouse strain used in scientific research, purification of MSC culture takes several weeks [25]. However, good results were obtained with a simple method, which started from plating the whole bone marrow aspirate (under culture conditions with 5% oxygen atmosphere, when available) and purification of MSC by a couple of passages, at weekly or longer intervals. Particularly in C57Bl/6 mice, we observed that the removal of CD45^pos^ cells took longer than in other strains and usually the first passage at which the culture was completely free of CD45^+^ cells was 6th passage. Once established in culture, the cells maintained their properties for at least 10 passages after the complete removal of hematopoietic cells.

In contrast to mouse MSC, human MSC can be obtained more rapidly from bone marrow [18]. The same is true for rat bone marrow, as well as from adipose tissue (of either species, human, mouse, rat) [26]. When these sources are used, MSC are readily obtained from the first passages, however, they become senescent and stop proliferating earlier (after around 4 passages for rat bone marrow and 6 passages for adipose tissue-derived cells) [27, 28].

The immunosuppressive capacity of MSC, irrespective of source of isolation (bone marrow or adipose tissue), appears to be a common feature of MSC among different species, although the mechanisms are varying from human to mouse. Thus, immunosuppression by human- or monkey-derived MSC is mediated by indoleamine 2,3-dioxygenase, whereas mouse MSC utilize nitric oxide, under the same culture conditions [29]. Direct comparison between the immunosuppressive capacities among different species was not addressed before and is likely difficult to estimate due to the heterogeneity of various MSC preparations, which endorses variability between experiments. However, it was reported that both mouse and human MSC ameliorated autoimmune diseases in syngeneic, allogeneic, and even xenogeneic settings [30], with no advantages provided by a particular source of isolation over the others. It is possible that the immunosuppression is not an intrinsic property of MSC, but is induced by inflammatory cytokines, which are released from activated T cells, as previously reported for human cells [29]. Corroborating to this, our data showed that the supernatant of MSC-activated T cell co-culture contained high levels of IFNγ, which ever exceeded the levels produced by activated T cells alone (Fig. 4c). This surge in IFNγ production by activated T cells in the presence of MSC was first reported by Cuerquis et al. in 2014 [31]. By demonstrating low levels if IL-2 and IL-10 synthesis in non-activated peripheral blood mononuclear cells (PBMC) in co-culture with MSC, as well as low levels of CD69 expression by T cells in the absence of proliferation, the authors made the suggestion that bone marrow-derived MSC induced a modest activation of initially resting T cells (the sub-population of T cells within the human PBMC) rather than a primary stimulatory full immune response followed with an inhibitory response [31].

### Controls

It is important to have CFSE-labeled unstimulated T cells as a control, as they provide the fluorescence median of non-dividing lymphocytes. This control will also help identifying the starting fluorescence peak (the parent population) in proliferating cells. The use of the CFSE-labeled T cells at time 0 to set the initial fluorescence level is not adequate, as the cells are extremely fluorescent within the first hours after staining and detection problems appear. Besides, the cells partially lose incorporated CFSE during the 3-day culture and consequently the CFSE-labeled resting T cells sample after 3 days in culture is the appropriate negative control for proliferation. Another important control is represented by unlabeled stimulated cells, in order to accurately set the autofluorescence level, knowing that the autofluorescence of proliferating lymphocytes is higher than that of resting lymphocytes.

### Comparison with other methods

Previous reports used CFSE-labeled lymphocytes to measure proliferation in the presence of proliferative MSC [29, 32–35]. The advantage of our protocol is given by the irradiation of MSC and the use of multiple doses, which allowed a more accurate dose-dependent estimation of the inhibitory effect. As in any co-culture setting in which the proliferation of one cell type (the responder cells) is to be measured, the non-proliferation of the other cell type (in our case, the inhibitor cells) is desirable. This number of MSC (inhibitory cells) totally inhibited the T cell proliferation (responder cell proliferation) under activating conditions. Consequently, a higher number of MSC would only produce an unnecessary cell crowding with a negative impact in the co-culture system (by nutrient depletion, pH changes, etc.), and no further benefits on the inhibitory effects. From hence, gradual decrease in the number of inhibitory cells in co-culture in the presence of a constant number of responder cells allows accurate determination of the inhibitory effect produced by MSC, by calculating the area under curve in the scatter plot representation of cell proliferation against MSC number. Our experiments showed an enhanced inhibitory effect of proliferative MSC, as compared to equivalent doses of irradiated cells. We assume that this data is the result of cell proliferation occurring during the 4 days of experiment (from the seeding day until the analysis day), which resulted in an increased MSC to T cell ratio by the end of experiment. Besides, in the presence of proliferative MSC, the dose-dependent inhibitory effect of MSC is lost and comparisons between different batches and/or conditions are not anymore feasible. This highlights the importance of mitotic inactivation of MSC in evaluating their immunosuppressive properties.

In a previous report, heat-inactivated MSC did not suppress T cell proliferation [36]. In contrast, our paper showed that co-culture of anti-CD3/CD28-activated T cells with irradiation-inactivated MSC strongly inhibited T cell proliferation in a dose-dependent manner. To evaluate the immunosuppressive capacity of MSC, it is important to have a standardized procedure that can follow lymphocyte proliferation with minimal disruption of cell viability. Our data showed that the viability of irradiated mouse MSC was not affected after 4 days of culture (Fig. 3c). The ^3^H-thymydine assay has become a gold-standard protocol to monitor rates of DNA synthesis and cell proliferation in mixed lymphocyte reaction experiments performed in vitro [37–39]. However, flow cytometry methods available today offer similar specificity and sensitivity with extra information in addition to the overall proliferative response [40–42]. The intracellular fluorescent dye, CFSE, has been found to be particularly effective at monitoring cell proliferation in vitro, as it is stably incorporated within cells and its fluorescence is sequentially splitting to half with each cell division [40].

A similar version of the protocol using human cells was reported in 2015. The group showed the dose-dependent inhibitory effect of several human MSC batches (obtained in three different Centers, with different protocols) on T cell sub-population from human peripheral blood mononuclear cells derived from different healthy donors [43]. Similar to the protocol described in our paper, the immunopotency assay was achieved in vitro; however, in the cited protocol hematopoietic cells were used after thawing from vapor phase-liquid nitrogen. Although murine MSC differ from human MSC in the mechanism of inhibition of T cell proliferation, being dependent on iNOS as upstream effector, as opposed to IDO, both mechanisms are using the same downstream pathways, namely endoplasmic reticulum stress [44]. Therefore, despite utilizing different means, immunosuppression of human T cells also involves cellular stress and thus it is a common strategy of immunoregulation conserved between mouse and humans. Furthermore, given the numerous evidences of the immunosuppressive potential of MSC in allogeneic and xenogeneic settings [45], this protocol can also be adapted for co-cultures of mouse splenic T cells with human MSC for evaluation of the immunosuppression activity of human cells.

### Biological significance

To date, there is no experimental procedure to directly evaluate the suppressive effects of MSC on T cell proliferation in vivo. This is partly owing to the fact that systemically delivered MSC rapidly accumulate in the lungs where they undergo apoptosis [46]. However, the in vivo impact of MSC is demonstrated in many animal experimental models of autoimmune diseases, in which significant decreases in the severity of disease are reported after syngeneic or allogeneic MSC infusion, suggesting that these cells modulate immune responses in vivo. Thus, human MSC administration to mice with established collagen-induced arthritis was accompanied by a reduced level of pathogenic CD4^+^T cells in the spleen and peripheral blood [47]. Another report showed the efficacy of syngeneic MSC as treatment of experimental autoimmune encephalomyelitis, mainly by inducing T cell anergy [48]. High-dose MSC transplantation in MRL/lpr mice (the murine model of systemic lupus erythematosus) effectively ameliorated disease activity by inhibiting abnormal activation of Akt/GSK3β signaling pathway of T cells [49]. Compelling evidence also demonstrated that MSC have unique capability to induce transplantation tolerance and inhibit the immune alloresponse at different levels, among which the decrease of effector T cells [50].

### Human relevance

The use of MSC in cell therapy is supported by the three important biological properties of these cells: (i) differentiation potential (mostly into mesoderm derivatives and only with low frequencies into cells of endodermal and ectodermal origin); (ii) secretion of protective factors (cytokines, proteolytic enzymes, and angiogenic molecules) that stimulate the proliferation and survival of endogenous cells within the local tissue, while inhibiting apoptosis and fibrosis; and (iii) immunoregulatory capacity. These are unique properties, which make MSC ideally suited for cell therapy for a wide range of diseases and disorders. Unfortunately, no in vivo assays are currently available to quantify either the multipotency or the immunosuppressive properties of MSC for a complete characterization. Therefore, distinguishing between different batches of MSC can only be done by in vitro assays.

As many other cell therapy products, MSC are susceptible to inherent heterogeneity as a result of differences between donors, tissue sources, and isolation protocols. Besides, the immunodulatory function of MSC was reported to depend on the degree of inflammation within the microenvironment [51]. As a result, cells generated in different studies may have differences in their biological properties and exert various degrees of immunosuppression in diverse pre-clinical and clinical settings.

Numerous evidences of the remarkable inhibitory effects of MSC on T cell proliferation in vitro and in vivo uphold MSC as suitable candidates in the treatments of autoimmune diseases, graft versus host disease, and allograft rejection. There is currently a large number of safety and efficacy clinical trials ongoing to investigate the use of MSC as a cellular immunotherapy [52]. For clinical applications, it is essential to start with a well-defined MSC population, including validated functionality. The protocol described in this paper can be used for the quantification of the immunosuppressive capacity of MSC on T cells. Additional studies may be warranted to determine how this in vitro assay correlates with other immunosuppressive properties of MSC, so that to predict the in vivo efficacy of each individual MSC batch.

## Conclusions

This paper presents a standardized protocol to assess the immunosuppressive effect of mouse MSC in vitro. The validation of the immunosuppressive capacity of MSC could be added to the characterization of the cells before being used in various MSC-based approaches to treat immunological diseases. Our results showed that mouse MSC have a dose-dependent inhibitory effect on activated T cell proliferation. The immunosuppressive properties of MSC vary between batches, but not between different passages of the same batch.

## Data Availability

The data that support the findings of this study are available from the corresponding author upon reasonable request.

## Abbreviations

MSC: Mesenchymal stem/stromal cells
CFSE: Carboxy-fluorescein succinimidyl ester
PI: Proliferation index
AUC: Area under the curve
iNOS: Inducible nitric oxide synthase
IDO: Indoleamine 2,3-dioxygenase
